# A nutrition-first, barrier-augmented, staged debridement management of grade IV oral mucositis in a leukemia patient with decompensated cirrhosis: A case report

**DOI:** 10.1097/MD.0000000000048943

**Published:** 2026-05-22

**Authors:** Yu Cheng, Youli Xue, Zhencheng Li, Hongxing Yang

**Affiliations:** a Nursing Department, University-Town Hospital of Chongqing Medical University, Chongqing, China; b College of Physical Education, Chongqing University, Chongqing, China.

**Keywords:** acute lymphoblastic leukemia, montmorillonite powder, multidisciplinary supportive care, oral mucositis

## Abstract

**Rationale:**

Oral mucositis (OM) is a common but serious complication of chemotherapy, particularly in patients with hematologic malignancies. Severe OM can significantly impair nutritional intake and quality of life, and may delay antileukemic therapy. This case report presents a multidisciplinary, integrative approach for managing grade IV OM in a patient with acute lymphoblastic leukemia and decompensated cirrhosis.

**Patient concerns:**

A 63-year-old female with a 13-year history of liver cirrhosis was admitted because of abdominal pain.

**Diagnoses:**

Laboratory tests revealed B-cell acute lymphoblastic leukemia, pancytopenia, and hypoalbuminemia. On day 15 of chemotherapy, oral mucosal ulcerations emerged and progressed to grade IV OM by day 29, characterized by hemorrhagic eschar, intolerable pain (numeric rating scale score 6), and complete inability to eat.

**Interventions:**

A multidisciplinary management strategy was implemented. Aggressive nutritional support was initiated, combining total parenteral nutrition with early enteral feeding via a nasogastric tube using a customized, blenderized formula enriched with branched-chain amino acids and whey protein. For wound care, hemorrhagic eschar was softened daily with saline, followed by cautious debridement to minimize pain and bleeding. Montmorillonite powder was applied to the wound to form a protective mucosal barrier and reduce infection risk. Pain management and psychological support were provided to enhance treatment adherence and maintain emotional stability.

**Outcomes:**

On day 63, oral mucositis had fully resolved, and antileukemic therapy proceeded without further interruption.

**Lessons:**

An integrated “nutrition-first, barrier-augmented, staged-debridement” management strategy may significantly enhance the healing of oral mucositis.

## 1. Introduction

Acute lymphoblastic leukemia (ALL) is a malignancy of lymphoid precursors and is associated with a poorer prognosis in adults compared with children.^[1]^ Current standard treatment still relies primarily on intensive multi-agent chemotherapy, which – while significantly improving survival – also carries substantial toxicity.^[2]^ Among these, oral mucositis (OM) is particularly common,^[3]^ arising from chemotherapy-induced epithelial injury and typically manifesting as painful inflammation and ulceration of the oral mucosa. Severe OM (grade III–IV [according to World Health Organization oral mucositis scale]) not only causes debilitating pain that prevents oral intake and compromises oral hygiene, but also markedly increases the risk of systemic infection and bleeding during profound myelosuppression. Consequently, OM frequently necessitates chemotherapy delays or dose reductions, thereby jeopardizing curative outcomes and substantially impairing patients’ quality of life.

Severe OM remains one of the most debilitating complications of chemotherapy in patients with ALL, often exacerbated by profound immunosuppression and systemic organ dysfunction.^[4]^ In these patients, neutropenia and thrombocytopenia significantly constrain the use of standard interventions such as debridement, while hepatic and renal impairment restrict safe administration of analgesics and antimicrobial agents. Moreover, the inability to maintain adequate oral intake perpetuates a catabolic state that impedes mucosal healing. These challenges are magnified in patients with complex comorbidities. The patient described in this case presented with an extraordinarily high-risk profile. In this case, conventional approaches were contraindicated due to a high risk of hemorrhage and impaired drug clearance. To address these limitations, montmorillonite was employed as a barrier-forming, hemostatic, and adsorptive topical agent – creating a stable wound microenvironment that enabled mucosal healing in the face of ongoing bleeding and infection risk.

Here, we present the case of an adult patient with ALL, decompensated cirrhosis, profound myelosuppression, and sepsis, who developed grade IV OM resulting in complete inability to tolerate oral intake. We describe a multidisciplinary management strategy that resulted in complete mucosal healing, resolution of pain, and timely resumption of both oral nutrition and antileukemic treatment. This report highlights a successful, hybrid approach to a life-threatening complication and offers a practical framework for managing severe OM in similarly high-risk hematology patients.

## 2. Methods

### 2.1. Ethical approval and informed consent statement

The institutional review boards of University-Town Hospital of Chongqing Medical University, where the case report was conducted, waived approval of the case report. The study was conducted in accordance with the Declaration of Helsinki. Informed consent has been obtained from the patient to publish this report. This is a case report.

## 3. Case report

A 63-year-old female with a 13-year history of liver cirrhosis was admitted to the hospital for abdominal pain lasting 1 day. She had previously been hospitalized in the Department of Gastroenterology and had been diagnosed with decompensated liver cirrhosis. On admission, physical examination revealed abdominal distension, ascites, and positive shifting dullness. Portal vein computed tomography angiography demonstrated esophageal–gastric varices and features of portal hypertension, along with imaging findings of liver cirrhosis and ascites, consistent with decompensated cirrhosis. Laboratory tests showed coagulation dysfunction, with elevated D-dimer (31.51 mg/L) and fibrin degradation products (86.3 mg/L), along with prolonged PT, elevated INR, prolonged APTT, and reduced fibrinogen, consistent with coagulopathy associated with advanced liver disease.

Laboratory tests revealed severe pancytopenia and hypoalbuminemia, with a white blood cell (WBC) count of 3.77 × 10^9^/L, red blood cell count of 2.30 × 10^12^/L, hemoglobin of 66 g/L, hematocrit of 0.20 L/L, platelet (PLT) count of 33 × 10^9^/L, total protein of 54.41 g/L, and albumin of 32.90 g/L (Table 1). Given the presence of pancytopenia, a bone marrow aspirate was performed. Flow cytometric immunophenotyping, conducted by an external certified laboratory, demonstrated findings consistent with B-cell acute lymphoblastic leukemia. Cytogenetic and molecular testing were not performed due to limited availability at the treating institution. Her numeric rating scale (NRS) score for pain was 2. The admission diagnoses included B-cell acute lymphoblastic leukemia, as well as decompensated liver cirrhosis with esophageal varices and portal hypertension, intrahepatic hemorrhage, and hypoalbuminemia.

**Table 1:** Dynamic changes in hematological parameters during chemotherapy.

Date	Day	WBC (×10^9^/L)	RBC (×10^12^/L)	Hb (g/L)	HCT (L/L)	PLT (×10^9^/L)	PCT (%)	Neu (%)	Lym (%)	Eos (%)	ANC (×10^9^/L)	ALC (×10^9^/L)	Eos (×10^9^/L)	CRP (mg/L)
2024/6/22	Day 1	3.77	2.3	66	0.2	33	–	85.4	10.4	–	–	–	–	22.15
2024/6/25	Day 4	4.98	2.12	61	0.18	20	–	94.4	–	–	–	–	–	31.61
2024/6/26	Day 5	2.27	2.29	65	0.2	24	–	89.2	–	–	–	–	–	–
2024/7/3	Day 12	0.11	2.57	76	0.22	6	–	–	–	–	0.03	–	–	11.69
2024/7/5	Day 14	0.25	1.59	47	0.14	0	–	91.3	–	–	0.23	–	0	144.15
2024/7/7	Day 16	0.19	2.73	82	0.24	6	0.01	–	–	–	0.11	0.07	0	112.28
2024/7/9	Day 18	0.76	2.68	81	0.24	24	0.02	78.1	10.5	0	0.59	0.08	0	39.78
2024/7/11	Day 20	9.9	2.35	71	0.21	31	0.04	92.5	4.5	0	9.15	0.45	0	19.72
2024/7/12	Day 21	9.9	2.35	71	0.21	31	0.04	92.5	4.5	0	9.15	0.45	0	19.72
2024/7/13	Day 22	14.8	2.51	77	0.23	103	–	96.4	1.2	0	14.26	0.18	–	16.48
2024/7/18	Day 27	8.47	–	–	–	–	–	93.2	–	–	—	–	–	51.7
2024/7/19	Day 28	9.25	–	74	–	323	–	92.9	–	–	8.59	–	–	–
2024/7/20	Day 29	7.87	–	76	–	347	–	92.8	2.7	0	—	–	–	28.91
2024/7/22	Day 31	8.01	–	80	–	301	–	93.4	–	–	7.48	–	–	21.62
2024/7/26	Day 35	5.02	–	81	–	166	–	81.3	9.5	0.1	—	–	–	–
2024/7/28	Day 37	6.53	2.85	93	0.28	143	–	79.7	13.3	0.2	—	–	–	9.61
2024/8/6	Day 46	12.87	–	90	–	103	–	–	–	–	9.12	–	–	21.04
2024/8/13	Day 53	6.82	–	83	–	83	–	41.5	–	–	—	–	–	53.01

ALC = absolute lymphocyte count, ANC = absolute neutrophil count, CRP = C-reactive protein, Eos = absolute eosinophil count, Eos = eosinophils (%), Hb = hemoglobin, HCT = hematocrit, Lym = lymphocytes (%), Neu = neutrophils (%), PCT = plateletcrit, PLT = platelet, RBC = red blood cell, WBC = white blood cell.

Chemotherapy was initiated immediately after hospital admission. On the same day, a peripherally inserted central catheter was placed to facilitate drug administration, and induction chemotherapy with the vincristine, daunorubicin, cyclophosphamide, and prednisone (VDCP) regimen was started. The VDCP regimen consisted of VDCP. Because the patient had preexisting liver disease, dose adjustments were considered when designing the chemotherapy protocol. Therefore, a reduced-intensity regimen was adopted, and the drug doses were further reduced by half during the later stage of treatment due to hepatic impairment. The dosing schedule was as follows: vincristine 2 mg on days 1 and 15; daunorubicin 60 mg/d on days 1 to 3; cyclophosphamide 600 mg on days 1 and 15; and prednisone 60 mg/d on days 1 to 14, followed by 30 mg/d on days 15 to 28.

On day 6 of hospitalization, the patient developed progressive somnolence accompanied by severe thrombocytopenia. Laboratory testing at that time demonstrated markedly elevated bilirubin levels, with total bilirubin of 92.16 μmol/L, direct bilirubin of 38.77 μmol/L, and indirect bilirubin of 53.39 μmol/L, indicating significant hepatic dysfunction. Liver function tests also showed hypoproteinemia and hypoalbuminemia (total protein 53.65 g/L, albumin 33.30 g/L) and elevated total bile acids (29.40 μmol/L). These findings, together with the patient’s history of decompensated cirrhosis, suggested hepatic encephalopathy as a possible cause of the altered mental status. A multidisciplinary consultation, including specialists from neurology, neurosurgery, and gastroenterology, was conducted. Based on clinical evaluation, intracranial hemorrhage was considered unlikely, and therefore brain imaging was not performed.

On day 12, her temperature increased to 38.7°C, and an infection was suspected. By day 14, her condition deteriorated with a fever of 38.1°C and profound myelosuppression. Serial blood tests demonstrated profound neutropenia following chemotherapy, the absolute neutrophil count (ANC) decreased to 0.03 × 10^9^/L, indicating severe neutropenia. The ANC remained critically low over the following days, measuring 0.23 × 10^9^/L on July 5 and 0.11 × 10^9^/L on July 7 (Table 1). She was transferred to the intensive care unit with a diagnosis of sepsis, requiring high-flow oxygen therapy and comprehensive supportive care, including broad-spectrum antibiotics, antifungals, and blood product transfusions.

The patient’s clinical course was complicated by the development of severe OM. On day 15, oral ulcerations were first noted and treated symptomatically. Despite initial management with topical solutions (Kangfuxin solution, Fucidin cream), the OM progressed. The patient was considered fit for transfer from the intensive care unit to the general ward on day 21 based on clear clinical and laboratory improvement (Table 1). Compared with the nadir observed around days 12 to 14 (WBC count 0.11–0.25 × 10^9^/L, ANC 0.03–0.23 × 10^9^/L, PLT count 0–6 × 10^9^/L), hematological parameters had significantly recovered by day 21 (WBC count 9.90 × 10^9^/L, ANC 9.15 × 10^9^/L, PLT count 31 × 10^9^/L). Inflammatory markers also decreased markedly (C-reactive protein reduced from 144.15 mg/L to 19.72 mg/L). In addition, liver function showed partial improvement, with declining bilirubin levels (from 92.16 μmol/L to 68.47 μmol/L) compared with earlier measurements. Based on these improvements and specialist evaluation by the hematology team, the patient was deemed clinically stable and no longer required intensive care monitoring. Therefore, transfer to the general ward was considered appropriate. She developed grade III OM with mucosal erosion, hemorrhage, and increased pain (NRS score 4). The condition continued to worsen, and on day 29, she had progressed to grade IV OM. The oral cavity and lips were covered in thick, dark eschar, with active bleeding upon movement. The pain intensified to an NRS score of 6, rendering her completely unable to tolerate oral intake. Consequently, after obtaining informed consent, a nasogastric tube was inserted to provide essential nutritional support.

Given the severity of the OM and its poor response to symptomatic treatments alone – which had substantially compromised her quality of life and impeded antileukemic therapy – we convened a multidisciplinary team to direct her management. The team comprised specialists from hematology, dermatology, clinical nutrition, gastroenterology, pharmacy, dentistry, and wound care nursing. Based on a literature review and expert consensus, an individualized, comprehensive management plan was formulated and implemented. The integrated management strategy included:

Nutritional support: crucially, this involved total parenteral nutrition supplemented by nasogastric feeding with a customized, blenderized diet enriched with branched-chain amino acids and whey protein to address severe hypoalbuminemia and malnutrition, with a target of energy approximately 25 to 30 kcal/kg/d and a protein intake of 1.3 to 1.5 g/kg/d.

Wound care and debridement: due to intense pain and high-bleeding risk (thrombocytopenia <10 × 10^9^/L), aggressive mechanical debridement was contraindicated. Instead, a stepwise approach was adopted. The eschar was softened with 0.9% saline-soaked gauze, followed by gentle removal of only loose fragments. This minimized bleeding and pain while gradually exposing the underlying wound bed.

Topical pharmacotherapy: a multi-agent topical regimen was applied sequentially after each cleaning. This included Gongfuxin solution to promote healing, bovine basic fibroblast growth factor spray (bFGF) for tissue regeneration, erythromycin ointment for bacterial prophylaxis, and a paste made from montmorillonite powder (Hunan Warrant Pharmaceutical Co., Ltd) to form a protective mucosal barrier. The paste was prepared by evenly applying montmorillonite powder over the wound surface, followed by covering it with saline-soaked gauze for approximately 10 minutes to allow adequate hydration. This topical regimen was applied 3 times daily.

Supportive care: this encompassed rigorous infection control protocols, pain management using topical lidocaine rinses before meals, and psychological support for the patient to alleviate anxiety and encourage adherence to the treatment plan.

**Figure 1.**
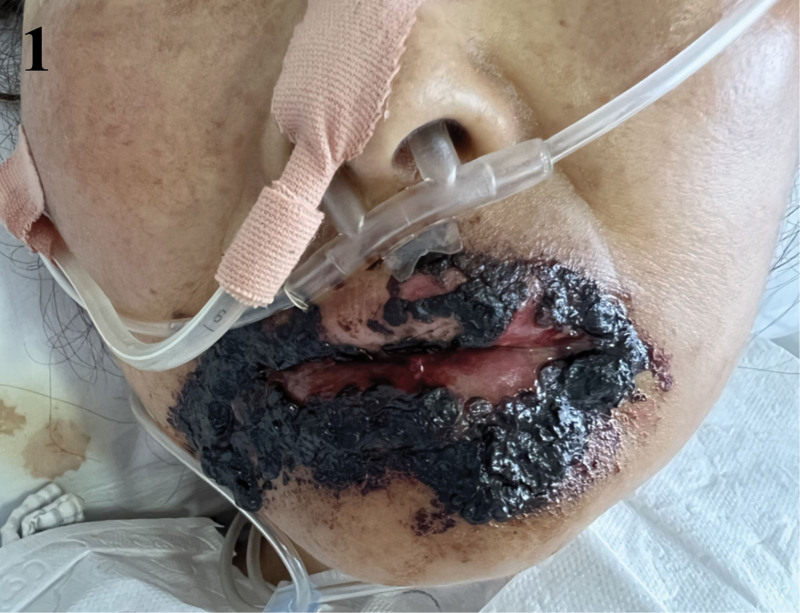
Severe hemorrhagic OM (WHO grade IV). WHO grade IV OM with diffuse hemorrhagic crusting and thick dark eschar over the lips and oral commissures; active bleeding; patient unable to tolerate oral intake. OM = oral mucositis, WHO = World Health Organization.

**Figure 2.**
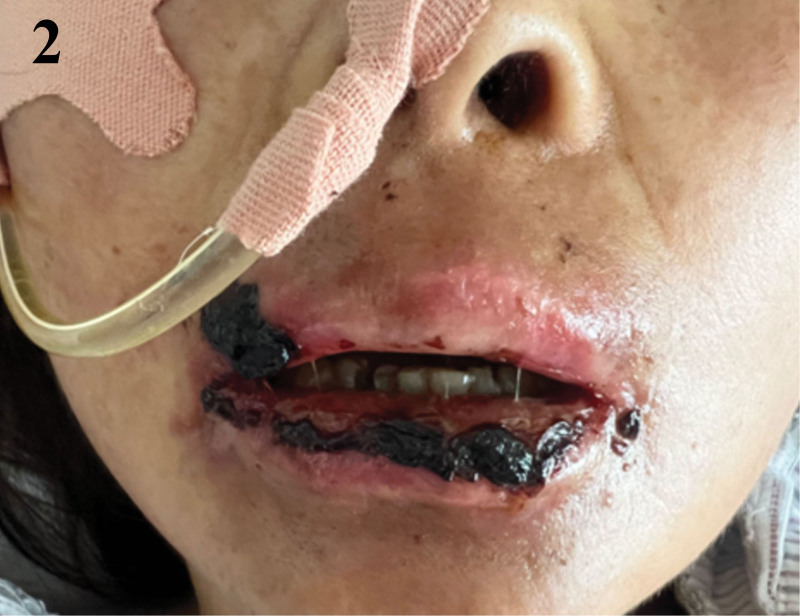
Early improvement after staged debridement (WHO grade III). WHO grade III OM after initiation of staged gentle debridement (saline softening with removal of loose eschar) and layered topical therapy (Kangfuxin rinse, bFGF spray, erythromycin ointment, montmorillonite paste); reduced eschar burden and improved hemostasis; liquid diet tolerated. bFGF = basic fibroblast growth factor spray, OM = oral mucositis, WHO = World Health Organization.

**Figure 3.**
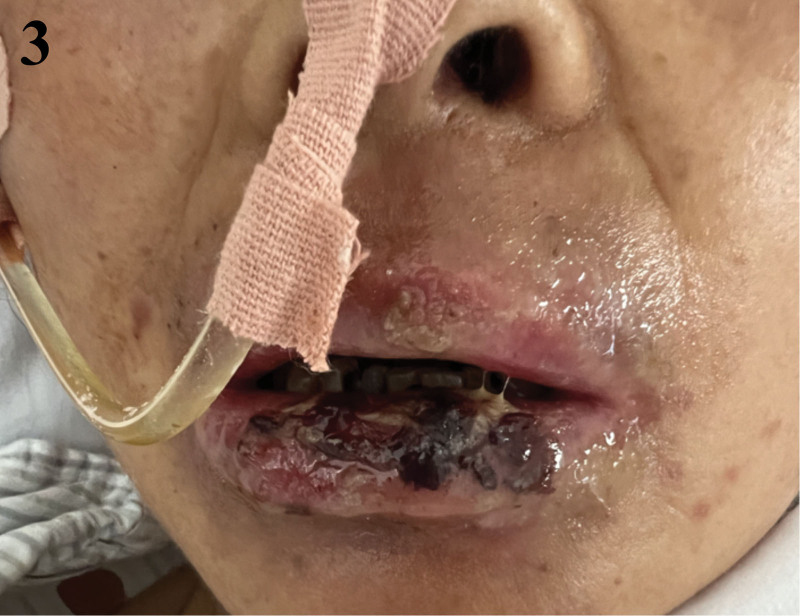
Ongoing mucosal healing. Improving grade III OM with decreased crusting and pain; continued liquid/soft diet with nasogastric supplementation. OM = oral mucositis.

**Figure 4.**
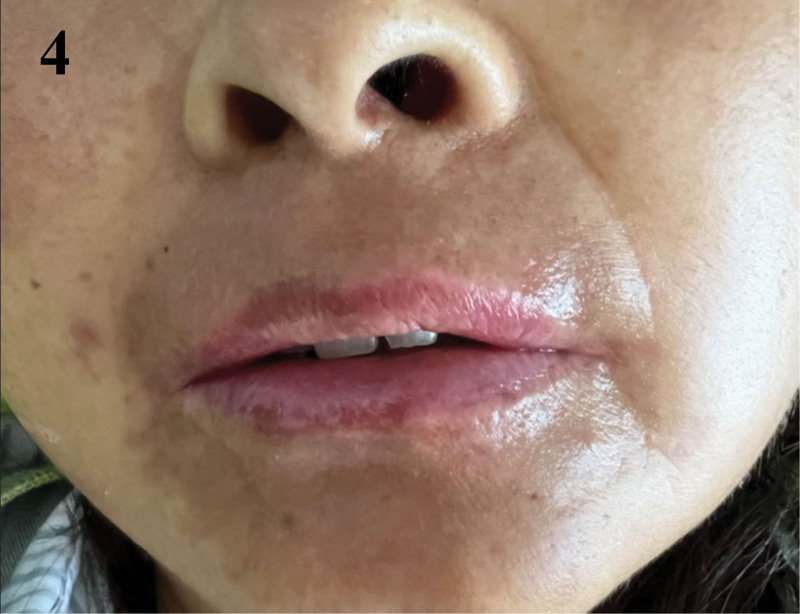
Near-complete resolution of oral mucositis. WHO grade I oral mucositis with near-complete reepithelialization and minimal residual erythema, pain resolved, and full oral feeding resumed. WHO = World Health Organization.

Following the implementation of this hybrid, multidisciplinary approach, the patient’s oral mucosa began to show signs of healing. On day 34, the patient still had World Health Organization grade IV OM with widespread hemorrhagic crusts (Fig. 1). A nontraumatic, stepwise debridement protocol was started – saline softening with removal of only non-adherent eschar – followed immediately by layered topical therapy (Kangfuxin rinse, bovine bFGF spray, erythromycin ointment, and montmorillonite paste as an adsorptive barrier). By day 41 the eschar burden had decreased and hemostasis improved; OM was downgraded to grade III, pain lessened, and a liquid diet was tolerated while nasogastric supplementation continued (Fig. 2). By day 49, there was further improvement within grade III with less crusting and pain (Fig. 3); oral intake advanced to liquid/soft, supported by nasogastric feeds to meet protein–energy goals. By day 63, near-complete reepithelialization (grade I) was observed with minimal residual erythema; pain resolved (NRS 0), full oral feeding resumed, and antileukemic therapy proceeded without delay (Fig. 4). No procedure-related hemorrhagic events occurred.

The clinical course showed that OM was most severe during the period of profound myelosuppression, corresponding to the nadir of neutrophil count (ANC 0.03–0.23 × 10^9^/L around days 12–14). The subsequent recovery of OM was temporally associated with hematologic recovery, particularly the increase in neutrophil and PLT counts from day 18 onward. Regarding treatment response, after the patient’s general condition improved, a repeat bone marrow evaluation was performed on July 29, 2024 (day 38). Bone marrow smear (day 39) demonstrated active granulopoiesis with reduced erythropoiesis. Flow cytometry (day 42) showed that CD34+ cells accounted for approximately 0.18% of nucleated cells, with no detectable abnormal immunophenotypic population. Minimal residual disease was <10^−4^, indicating complete remission. Based on these findings, there was clear evidence of a response to chemotherapy. This supported the decision to resume chemotherapy after recovery from OM. The treatment plan was to continue the chemotherapy regimen, and the patient subsequently completed further chemotherapy as scheduled.

## 4. Discussion

This case highlights 3 pivotal management decisions that, in concert with standard supportive care, enabled reversal of grade IV OM in a profoundly immunocompromised patient with ALL, decompensated cirrhosis, and sepsis.

First, in the setting of severe thrombocytopenia and coagulopathy leading to oozing from oral ulcerations, the application of montmorillonite paste as an adsorptive mucosal barrier demonstrated clear clinical benefit. Montmorillonite is commonly used in clinical practice as an oral antidiarrheal agent or a mucosal protectant.^[5,6]^ Its therapeutic effects are attributed to its strong adsorptive capacity, ability to form a protective barrier over mucosal surfaces, and modulation of the local microenvironment, thereby reducing irritation and promoting mucosal repair.^[5]^ Previous reports have also described its application in wound hemostasis.^[7]^ For example, surgical procedures involving intra-abdominal adhesions are often associated with a high risk of bleeding,^[8]^ and montmorillonite has been used as a hemostatic agent in such settings.^[9]^ In the present case, montmorillonite application facilitated a cleaner wound bed, thereby allowing other topical agents (e.g., bFGF spray, antibiotic ointment) to act more effectively. The use of montmorillonite was temporally associated with stabilization of mucosal bleeding and relief of pain. We acknowledge that this represents off-label use and that causality cannot be inferred; nevertheless, these observations suggest a potential adjunctive role for clay-mineral barrier formulations, such as montmorillonite, in the management of high-bleeding-risk OM.

In this case, the patient’s decompensated liver cirrhosis posed a unique challenge for nutritional management.^[10]^ Malnutrition and muscle wasting are very common in advanced cirrhosis and can in fact exacerbate hepatic encephalopathy.^[11]^ Modern guidelines therefore advise against protein restriction even in cirrhotic patients with encephalopathy; instead, a high-protein intake (approximately 1.2–1.5 g/kg/d) is recommended to promote positive nitrogen balance and support recovery.^[11]^ Early provision of high-protein nutritional support was thus deemed essential for this patient – not only to counteract the catabolic state of liver failure, but also to promote the healing of her oral ulcers by creating an anabolic environment conducive to tissue repair.^[12]^ Moreover, compared with exclusive parenteral nutrition, enteral nutrition is advantageous in preserving gastrointestinal integrity and reducing the risk of bacterial translocation and infection. Infection control was a critical priority for our patient, since her condition was associated with immune dysfunction and any infection could precipitate worsening treatment courses. Thus, despite the potential bleeding risks of nasogastric tube placement (e.g. variceal hemorrhage in cirrhosis), an “enteral-first” strategy was adopted. In practice, using a soft, small-bore nasoenteric feeding tube minimizes trauma; notably, such tubes have not been shown to significantly increase variceal rebleeding risk in patients with esophageal varices.^[13]^

Third, a staged debridement strategy was employed. Saline-soaked gauze was first applied to soften thick hemorrhagic eschar, followed by removal of only loose fragments, with the key objective of enabling topical drug delivery to the ulcer bed while minimizing bleeding and pain. In OM associated with coagulopathy, aggressive mechanical debridement is generally contraindicated^[14]^; however, in this case, a conservative approach combined with immediate compressive hemostasis and layered topical therapy (healing promoter + antibacterial ointment + barrier paste) facilitated gradual reepithelialization and improved mouth opening without precipitating major hemorrhage. This approach may provide a practical management option when surgical debridement is not feasible.

Taken together, the 3 interventions in this case – barrier-focused topical care with montmorillonite, early initiation of enteral nutrition despite bleeding risk, and staged conservative debridement – acted synergistically within a multidisciplinary bundle that also included analgesia, infection control, and psychological support. These findings should be interpreted with caution: this is a single-patient experience, and neutrophil recovery, ongoing antimicrobial therapy, and other concurrent interventions likely contributed to healing, making the relative effect of each component indeterminate. Nevertheless, the reproducible logic of this sequential strategy, along with the favorable symptomatic improvement observed in a neutropenic patient, suggests potential clinical relevance and warrants further investigation.

## Acknowledgments

The authors used OpenAI ChatGPT (GPT-5 Thinking) to assist with language editing and formatting of this manuscript. The tool did not generate or modify raw clinical data, diagnoses, or management decisions, and was not used for statistical analysis or for automated reference generation. Only de-identified clinical information was provided to the tool. All AI-assisted outputs were critically reviewed, revised, and approved by the authors, who take full responsibility for the accuracy, integrity, and ethical compliance of the final manuscript.

## Author contributions

**Conceptualization:** Yu Cheng.

**Data curation:** Youli Xue.

**Validation:** Youli Xue.

**Writing – review & editing:** Youli Xue, Zhencheng Li, Hongxing Yang.

**Writing – original draft:** Zhencheng Li.
